# Transverse spinal cord infarction following immunoglobulin treatment in a patient with exfoliative dermatitis: A case report

**DOI:** 10.1097/MD.0000000000037719

**Published:** 2024-04-12

**Authors:** Lili Zhang, Lanying He, Jing Huang, Sixie Ren, Jian Wang

**Affiliations:** a Department of Neurology, The Second People’s Hospital of Chengdu, Chengdu, China; b Department of Medical Imaging, The Second People’s Hospital of Chengdu, Chengdu, China

**Keywords:** Aortic thrombosis, intravenous immunoglobulin, pulmonary embolism, spinal cord infarction, thrombotic events

## Abstract

**Rationale:**

Transverse spinal cord infarction (SCI) is rare but highly disabling. Aortic thrombosis was described as one of the most common etiologies. Thromboembolic complications associated with intravenous immunoglobulin (IVIG) have been reported.

**Patient concerns:**

A previously well, 64-year-old man who was given the treatment of IVIG (0.4 g/kg/d for 5 days) for exfoliative dermatitis 2 weeks before, progressively developed flaccid paraplegia of lower extremities, loss of all sensations below T3 level and urinary incontinence within 50 minutes.

**Diagnoses:**

A diagnosis of SCI and pulmonary embolism was made. IVIG was considered the possible cause.

**Interventions:**

Anticoagulation treatment and continuous rehabilitation were administered.

**Outcomes:**

The neurologic deficiency of the patient was partially improved at the 3-year follow-up.

**Lessons:**

The rapid development of severe deficits within 4 hours mostly contributes to the diagnosis of SCI. Heightened awareness of possible thrombotic events is encouraged for a month-long period following IVIG therapy.

## 1. Introduction

Transverse spinal cord infarction (SCI) is a rare but highly disabling central nervous disorder.^[1,2]^ Aortic thrombosis was described as one of the most common etiologies.^[3]^ Intravenous immunoglobulin (IVIG), widely used in the treatment of autoimmune diseases, is considered relatively safe while serious thromboembolic complications have been reported in sporadic cases.^[4]^ So far none of the cases regarding segmental artery thrombosis causing SCI have been reported. Herein, we describe a case of transverse SCI with aortic thrombosis following IVIG therapy for exfoliative dermatitis.

## 2. Case presentation

**Figure 1.**
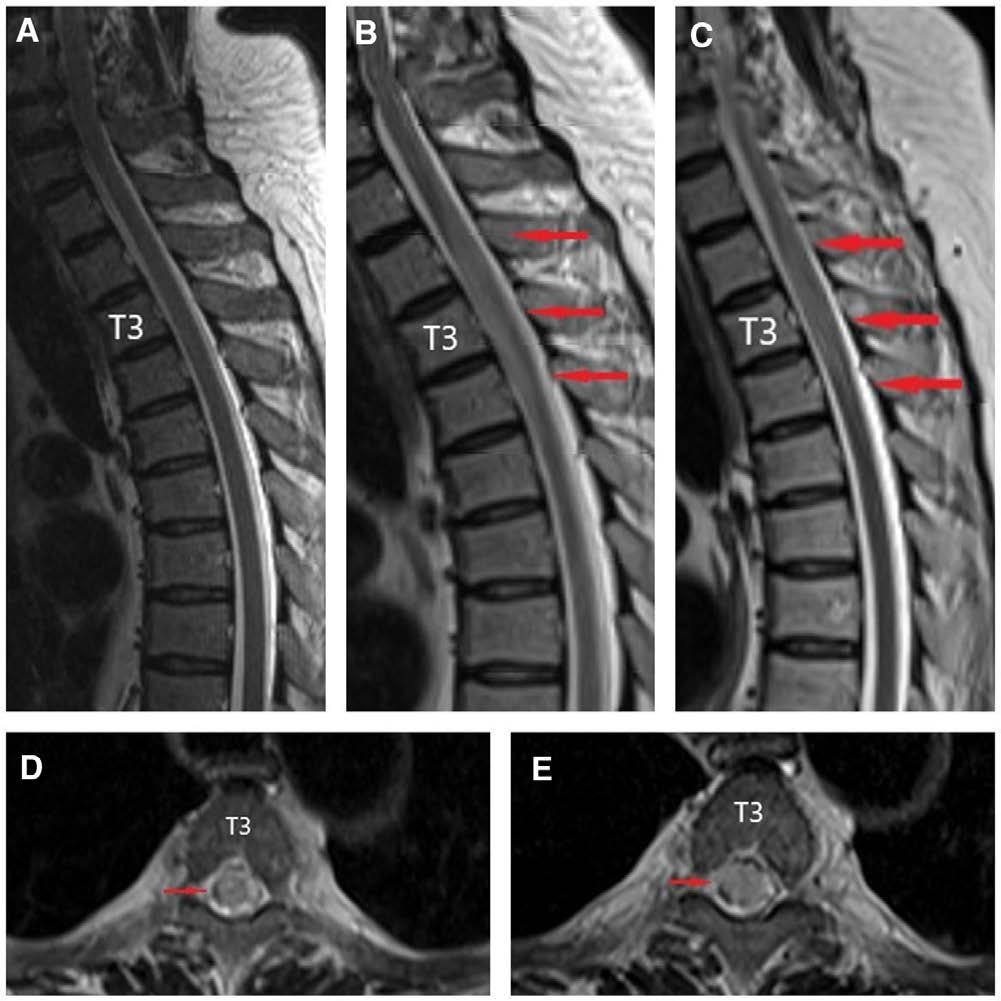
(A) MRI of the thoracic vertebra showed no cord injuries. Sagittal (B) and Axial (D) MRI of the thoracic vertebra demonstrated slight thickening of the 3rd to 5th thoracic spinal cord with increased T2 signal (red arrow). Sagittal (C) and Axial (E) MRI of the thoracic vertebra confirmed no obvious change compared with imaging in (B and D). MRI = magnetic resonance imaging.

**Figure 2.**
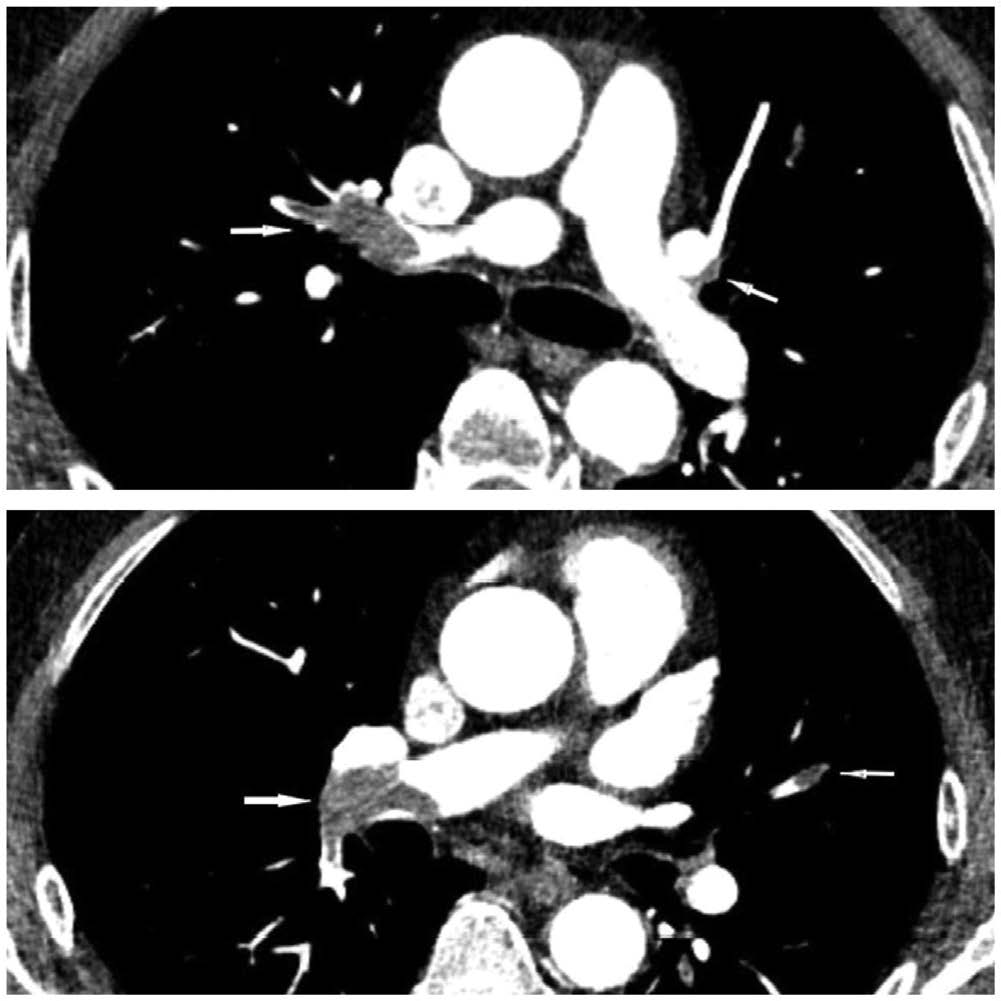
CTA of thoracic aorta showed bilateral multiple pulmonary embolism (white arrow). CTA = computed tomography angiography.

**Figure 3.**
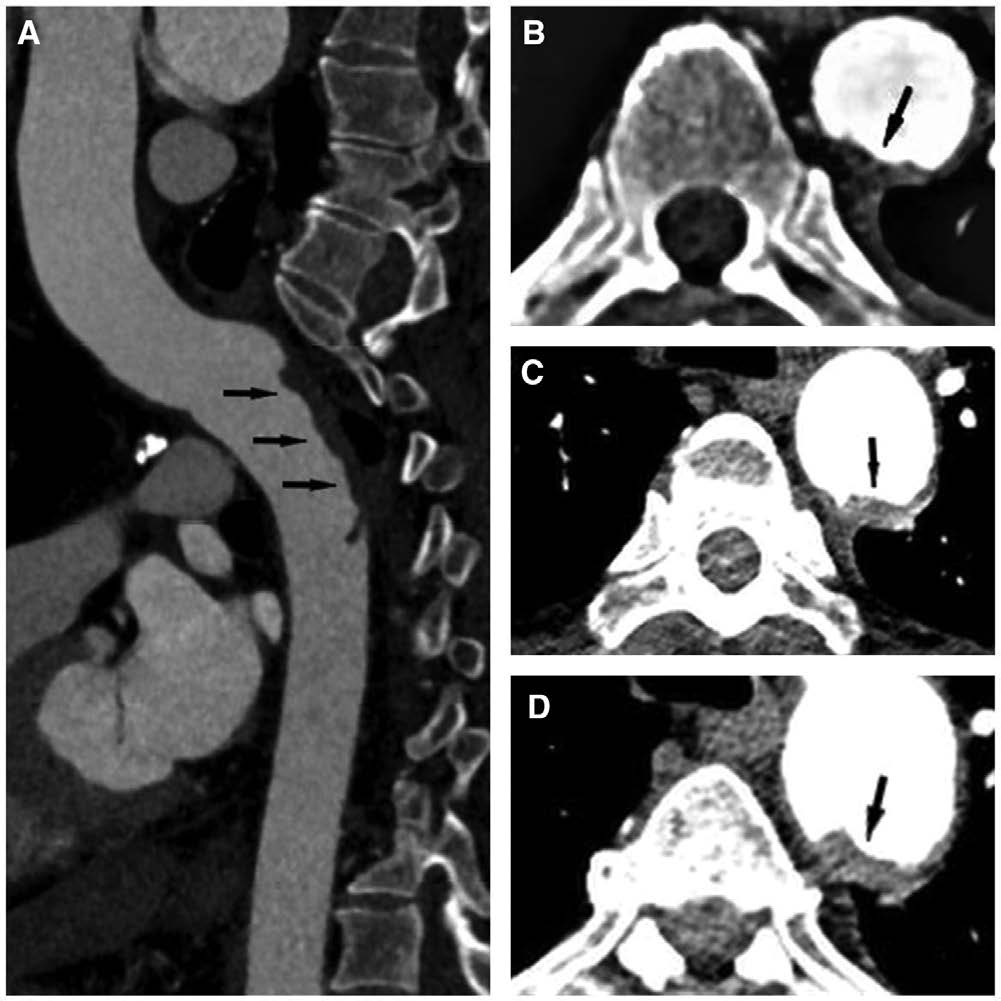
CTA of thoracic aorta on sagittal (A) and axial (B–D) views displayed descending aorta thrombosis at the level of thoracic vertebral bodies from T4 to T6 (black arrow). CTA = computed tomography angiography.

A previously well, 64-year-old man was given the treatment of intravenous methylprednisolone (40 mg bid for 8 days, then 60 mg qd for 6 days) and human immunoglobulin (0.4 g/kg/d for 5 days) for exfoliative dermatitis 2 weeks before, progressively developed flaccid paraplegia of lower extremities, loss of all sensations below T3 level and urinary incontinence within 50 minutes. American Spinal Injury Association (ASIA) grade A was graded. Tests for antinuclear antibody, MPO-ANCA, PR3-ANCA, antiphospholipid antibody, lupus anticoagulant, protein C/S, oligoclonal band, AQP4, and MOG were negative. Cerebral spinal fluid analysis showed normal opening pressure (108 mm H_2_O), white cell count (1 cell/μL), protein (26.8 mg/dL), and glucose level (64 mg/dL). The d-dimer level elevated remarkably from 0.91 to 23.7 μg/mL 2 days later. Emergent magnetic resonance imaging (MRI) of the thoracic vertebra suggested no evidence of cord injuries (Fig. 1A). However, a repeat MRI scan after 2 days revealed a transverse spinal cord lesion with associated edematous T2-hypertensity extending from T3 to T5 (Fig. 1B, D). A thoracic computed tomography angiography (CTA) revealed bilateral multiple pulmonary thromboembolism (Fig. 2) and the descending aorta thrombosis leading to the obstruction of the segmental branches off the aorta (Fig. 3).

## 3. Treatment

A diagnosis of SCI and pulmonary embolism was made. Then anticoagulation treatment with low molecular weight heparin (LMWH) (6000IU Q12h) followed by rivaroxaban and inpatient rehabilitation were administered. A 3rd MRI performed 1 month after symptom onset showed the T2 hyperintensity persisted (Fig. 1C and E). No neurologic deficiency improvement was observed except for the sense of pain and touch below level T3 at the 3-year follow-up.

## 4. Discussion

The definite diagnostic criteria of SCI proposed by Zalewski et al^[5]^ were met in this patient. The etiology of thoracic medullary infarction was primarily extensive intraluminal thrombus of the descending aorta leading to the occlusion of thoracic radicular arteries with the lesion appearing in the longitudinal watershed areas at level T3 to T5.^[6]^ Considering the lesions exhibited in both the arterial and venous circulation, we thought that aortic thrombosis rather than atherosclerosis could be the main etiology of SCI.

The source of the embolus is unclear. It might be associated with the previous IVIG. As reported, most side effects of IVIG are mild and transient, and serious complications are rare and mainly occur in patients with high-risk factors.^[4]^ Thrombotic events as serious delayed adverse effects of IVIG have been identified in some cases.^[4,7]^ The thrombosis site is arterial in 80% of reported cases including stroke and myocardial infarction that occur within hours or days of an infusion. Venous thrombosis containing deep vein thrombosis, and pulmonary embolism generally occurs in 20% of cases days or weeks after an infusion.^[8]^ In our case, SCI and pulmonary embolism occurred following IVIG and might be potentially triggered by hyperviscosity, the activation of procoagulant factors, autoimmune vasculitis, or vasospasm.^[4]^ Immunoglobulin half-life can be up to 18 to 32 days, and high concentration persists for several days after infusion,^[9]^ which possibly in part, explains why it took 9 days for IVIG-associated thrombosis to happen in our patient. Besides, the absence of preexisting atherosclerosis or prior embolic events supports a primary thrombogenic role of IVIG.

However, IVIG is a therapeutic biologic agent that has been prescribed for over 2 decades to treat various neurological and neuromuscular conditions. Large researches and studies suggested the efficacy of IVIG in several autoimmune disorders,^[10]^ as Guillain–Barré syndrome, chronic inflammatory neuropathies, refractory exacerbations of myasthenia gravis, Lambert–Eaton syndrome, dermatomyositis, stiff person syndrome, and so on. Treatment with IVIG must be administered in the context of its known adverse effects. Given that, individualized treatments depending on patient needs and physician judgment are important.

MRI is the most valuable diagnostic tool when evaluating spinal cord syndromes but is not sensitive, especially in the early course of symptom onset. Thus, it is paramount to repeat an MRI scan days later.^[11]^ However, emergent MRI is vital to rule out mimics that require acute management. MRI abnormalities including cord swelling and hyperintensity on T2-weighted images may be seen.^[12]^ In terms of our patient, a transverse T2-hypertensity lesion of T3 to T5 was found on MRI after 2 days from onset. Diffusion-weighted imaging can be valuable in the diagnosis of SCI. Nonetheless, it is limited by initial insensitivity resulting from lower spatial resolution and greater susceptibility artifact.^[2,5]^ It is not routinely performed. CTA can be performed to look for etiologies of SCI. Thoracic CTA helped to identify the aortic thrombus as the cause of cord infarction in this case.

Importantly transverse myelitis needs to be differentiated from SCI. A hyperacute presentation indicates SCI, while a subacute presentation suggests an inflammatory cause.^[13]^ The lower time limit for the nadir of maximum deficits to diagnose transverse myelitis was given with 4 hours.^[14]^ The MRI findings of SCI are usually normal during the initial 24 hours. Whereas, patients with inflammatory disease have abnormal MRI features at an early stage.^[12]^ Diffusion-weighted imaging can further help in the diagnosis of SCI.

The quality of evidence for SCI treatment is currently weak. Intravenous tissue plasminogen activator was proved ineffective in SCI patients.^[5,15]^ It was reported that vasopressors could increase the spinal cord perfusion pressure through the collateral circulation.^[16]^ Other treatment includes antithrombotic therapies (antiplatelet or anticoagulation). In spite of increasing thrombotic risk, empirical corticosteroids can be administered when a possible inflammatory myelopathy is concerned.^[5]^ Plasma exchange which can reduce cord perfusion^[17]^ and intravenous immunoglobulin which is prothrombotic^[18]^ should be avoided.

## 5. Conclusion

We encountered a rare case of transverse SCI and pulmonary embolism which was possibly related to immunoglobulin administration for exfoliative dermatitis. The rapid development of severe deficits within 4 hours mostly contributes to the diagnosis of SCI. Heightened awareness of possible thrombotic events is encouraged for a month-long period following IVIG therapy.

## Author contributions

**Writing—review & editing:** Lili Zhang, Sixie Ren.

**Writing—original draft:** Lanying He.

**Supervision:** Jing Huang, Jian Wang.
